# The Influence of Sleep and Autonomic Recovery on Performance in World-Class Swimmers at Major Competitions: The Qualifying Trials, World Championships, and Olympic Games

**DOI:** 10.3390/sports14100426

**Published:** 2026-10-01

**Authors:** Lidia Barragán, Carmen Crespo, Sergio J. Ibáñez, Rafael Timón, Guillermo Olcina

**Affiliations:** 1 Group for Advances in Sports Training and Physical Conditioning (GAEDAF), Faculty of Sports Sciences, University of Extremadura, 1003 Caceres, Spain; ccrespoc@unex.es (C.C.); rtimon@unex.es (R.T.); golcina@unex.es (G.O.); 2 Sports Training and Performance Optimization Group (GOERD), Faculty of Sports Sciences, University of Extremadura, 1003 Caceres, Spain; sibanez@unex.es

**Keywords:** sleep efficiency, heart rate variability, resting heart rate, Olympic Games, elite swimmers, wearable technology

## Abstract

Sleep deprivation is one of the main risk factors for impaired performance in elite athletes, but the changes in sleep and autonomic regulation in response to competitions of varying levels within the same Olympic year remain unknown. In this prospective, longitudinal, observational study, we monitored parameters related to sleep quality and quantity, as well as heart rate variability and resting heart rate, in a cohort of eight Olympic swimmers (*n* = 8) during the World Championships in Doha, the qualifying trials, and the 2024 Paris Olympic Games, using the Oura Ring Gen3 under real-world conditions throughout the pre-competition, competition, and post-competition phases of each event. The Olympic Games were the only event where sleep efficiency fell below the commonly used reference threshold of 85% during the competition phase accompanied by a reduction in the total sleep score and an increase in resting heart rate. The Olympic setting was accompanied by temporary changes in sleep and autonomic markers, but no sleep parameter or autonomic marker showed a statistically significant association with intra-individual competitive performance, although the limited sample size constrains the power to detect such relationships.

## 1. Introduction

Sleep is an active metabolic state essential for the physiological recovery of elite athletes. During nighttime rest, motor memory consolidation [1], tissue repair [2], and restoration of homeostasis [3] occur. However, up to 64% of elite athletes report poor sleep quality on the nights leading up to major competitions, with a higher prevalence in individual sports that lack the collective buffering of stress [4]—a pattern attributed to the shared distribution of responsibility for the outcome among teammates, which reduces individually perceived pressure compared with sole accountability in individual sports [5]. Consistent with this, athletes from individual sports go to bed earlier, wake up earlier, and obtain less sleep (6.5 h vs. 7.0 h) than athletes from team sports [6]; both groups sleep below the recommended 8 h, and individual-sport athletes report greater difficulty falling asleep the night before important competitions [5,6]. These disturbances affect not only total sleep duration but also its internal architecture, compromising the deep sleep (N3) and rapid eye movement (REM) phases, which are essential for neuromuscular recovery [7], endocrine regulation, and emotional stability in highly demanding contexts [8]. In elite swimming, the culture of early-morning training sessions severely restricts the total sleep time (TST) of swimmers, an effect directly attributable to the training schedule [9], while the combination of morning heats and evening finals in major championships further fragments the sleep–wake cycle during the most critical phases of the competitive calendar [10].

Beyond the amount of sleep, heart rate variability (HRV) and resting heart rate (RHR) have emerged as autonomic markers that are sensitive to an athlete’s internal load. A sustained reduction in vagal HRV has been associated with states of overload, although the relationship with training load and fitness in elite athletes is non-linear and can be equivocal [11]. Moreover, unexpected increases in RHR may suggest accumulated fatigue, persistent sympathetic activation [12], or incipient illness, although autonomic responses to overreaching can vary and sometimes present as reduced RHR with vagal predominance [10,11]. Integrating both markers with objective sleep metrics provides a multidimensional approach to assess recovery status, which is particularly relevant in individual sports such as swimming, where there is no collective buffering of competitive stress. The emergence of wearable devices such as the Oura Ring—whose concordance with polysomnography (PSG, the clinical gold standard [13]) has been evaluated for global sleep variables [14], albeit with acknowledged limitations in phase staging—has made this type of monitoring feasible under real-world conditions [15], overcoming the logistical barrier that has historically limited research in this field.

Despite the accumulated evidence, existing studies have focused predominantly on a single phase or isolated competition, without addressing how sleep parameters and autonomic regulation evolve over the course of a full Olympic year with events of varying importance [12,13]. In particular, it is unknown whether the differential impact of the Olympic Games on sleep translates into distinct autonomic patterns and whether any of these markers predict intra-individual competitive performance. Therefore, the primary objective of this study was to longitudinally assess sleep parameters and autonomic physiological markers in Olympic swimmers across three competitions of varying levels in the 2024 Olympic year: the World Championships in Doha, the qualifying trials, and the Paris Olympic Games. As a secondary, exploratory objective, we examined the association between these markers and intra-individual competitive performance.

## 2. Materials and Methods

### 2.1. Participants

Eight Olympic swimmers (5 women and 3 men; mean age 21.2 ± 2.9 years, range 18–26 years) were included in the study, selected from the national swimming programme in both pool and open-water disciplines. All participants were of Caucasian ethnicity, corresponding to Fitzpatrick skin type III [16]. This sample represented 44.4% of all federated Olympic swimmers in Spain during the 2023–2024 cycle (*n* = 18). While this proportion is high relative to the national population, generalisability beyond this specific cohort remains limited by the small absolute sample size (*n* = 8). The participants were classified at Tier 5, the highest level of international competitive performance, and all had prior experience in national and international competition [17]. As members of the national high-performance programme managed by the RFEN, the cohort represents a substantial proportion of Spanish swimmers competing at Olympic level during the 2023–2024 cycle. Participants were identified and contacted through the Spanish Swimming Federation (RFEN), which manages the national high-performance centres to which all eligible swimmers belonged. Eight athletes were invited to participate, and all eight met the eligibility criteria and completed the monitoring protocol. Athletes were eligible if they were part of the national high-performance programme, had participated in national or international competitions, and were able to complete the protocol. Athletes with active musculoskeletal injuries or medical conditions that limited regular participation in training or competition, or those who completed less than 80% of the monitoring protocol entries, were excluded. There were no exclusions.

### 2.2. Ethical Considerations

This study was conducted in accordance with the principles of the Declaration of Helsinki and was reviewed and approved by the Bioethics and Biosafety Committee of the University of Extremadura (ref. 82/2025). Prior to inclusion, all participants received detailed information about the study’s objectives, procedures, and potential risks, and signed the corresponding informed consent form.

### 2.3. Study Design

This study used a prospective, longitudinal, observational design with repeated measures, in which each participant served as their own control. Following the multidimensional classification proposed for sports science [18], it corresponds methodologically to an associative design. Data collection took place in a real-world ecological context, with a high degree of protocol standardization (a validated wearable device and predefined time windows) but no control over training or competition conditions, consistent with applied high-performance sport research. The unit of analysis was the individual swimmer; fitting linear mixed models with subject as a random effect combined an idiographic approach—each athlete serving as their own control—with a nomothetic approach at the group level (*n* = 8). The follow-up spanned three major international competitions in the 2023–2024 Olympic cycle: the World Championships in Doha (February 2024), the Qualifying Trials (June 2024), and the Paris Olympic Games (July–August 2024), each divided into three phases: pre-competition (the seven days before the event), competition (standardized seven-night monitoring window encompassing the competition period and associated travel), and post-competition (the seven days following the event’s end).

### 2.4. Implementation

Sleep and physiological parameters were monitored using the Oura Ring Gen3 (Oura Health Oy, Oulu, Finland), a non-invasive smart ring that combines infrared photoplethysmography, triaxial accelerometry, and peripheral skin temperature sensors to continuously estimate sleep parameters and autonomic activity. Sleep staging was derived using the Oura Sleep Staging Algorithm 2.0 (OSSA 2.0) [19], with device firmware automatically updated by the manufacturer throughout the monitoring period (versions 2.9.24–2.9.33). Its validity compared with PSG, the clinical gold standard, is supported by recent research. One study found no significant differences in TST or time in bed (TIB) between the Oura Ring Gen3 and PSG, with an overall accuracy of over 91% [19]. In another study, researchers reported a Pearson correlation (r) of 0.86 for TST compared with actigraphy [20]. For the autonomic markers analysed in the present study, a validation study comparing the Oura Ring Gen3 against a reference electrocardiogram reported strong agreement for both nocturnal RHR (Lin’s concordance correlation coefficient = 0.97; mean absolute percentage error = 1.67%) and HRV/rMSSD (concordance correlation coefficient = 0.97; mean absolute percentage error = 7.15%) [21]. Regarding device suitability across skin tones, a free-living validation study of the Oura Ring Gen3 across the full Fitzpatrick scale (types 1–6) found no significant association between sleep-tracking error and skin tone [22].

The recordings were automatically synchronised with the official Oura Teams platform (Oura Health Oy, Oulu, Finland), accessible via personal credentials from the web version, from which the data corresponding to each time window were extracted for subsequent analysis.

### 2.5. Variables

Six dependent variables were analysed. The following were used as indicators of sleep quality and quantity: the total sleep score (SS; 0–100 scale), sleep efficiency (SE; %), TIB (hours), and TST (hours). HRV (ms) and RHR (beats per minute [bpm]) were used as autonomic physiological markers. HRV was quantified using the root mean square of successive differences (rMSSD), the standard time-domain metric provided by the Oura platform, calculated as the average of the 5-min rMSSD samples recorded throughout the night. RHR corresponded to the average RHR recorded during the night, as provided by the Oura platform.

Competitive performance was determined based on each athlete’s official time in their specialty event at each competition. Because the participants competed in heterogeneous events, the comparison was conducted on an intra-individual basis: each swimmer was compared only to their own performance across the three competitions, using their best time from the three events (Level 1), their intermediate time (Level 2), and their relatively worst time (Level 3) as reference points. This ordinal classification was adopted because the heterogeneity of swimming events across participants (ranging from short pool distances to open-water races) precluded direct comparison of absolute time differences at the group level; ranking each swimmer’s own three performances provided a consistent, event-independent basis for group-level analysis. This approach, however, does not capture the magnitude of the difference between performances, which may vary substantially between swimmers and across transitions. As a complementary continuous measure, intra-individual performance change was also expressed as the percentage change in official time between consecutive competitions [(time_2_ − time_1_)/time_1_ × 100], calculated separately for the World Championships-to-Trials and Trials-to-Olympic Games transitions; negative values indicate a faster (improved) time.

### 2.6. Procedure

Each participant wore the Oura Ring Gen3 continuously throughout the competitive season, from the start of the competitive period at the World Championships in Doha (2 February 2024) until the end of the post-competition period at the Paris Olympic Games (11 August 2024). The ring was removed only during official competition events, in accordance with World Aquatics regulations, and was worn during training sessions and all other daily activities. Compliance with nighttime wear was based on the athlete’s self-reporting and was verified retrospectively through the continuity of data on the Oura platform. Each phase (pre-competition, competition, and post-competition) comprised seven nights of monitoring per event, yielding 504 expected nights of data across the three competitions and eight swimmers (7 nights × 3 phases × 3 competitions × 8 swimmers). All participants adhered to the protocol during the analyzed time periods, with no data gaps in any period, resulting in 504 completed nights of monitoring. Performance data were obtained from the official records of the organising federations (National Federation, World Aquatics, and the International Olympic Committee).

### 2.7. Statistical Analysis

The data are expressed as the mean ± standard deviation. Although univariate normality and homogeneity of variance were confirmed for all variables and phases (Shapiro–Wilk and Levene’s tests, respectively, *p* > 0.05), multivariate normality was not met (Shapiro–Wilk W = 0.767, *p* < 0.001). Therefore, Pillai’s trace was selected as the primary multivariate test statistic due to its robustness to departures from normality. The significance level was set at α = 0.05. Given the small sample size (*n* = 8), effect sizes were used as the primary indicator of practical significance. Specifically, partial eta-squared (η^2^p) is reported alongside its 90% confidence interval (CI) for the main effects, interpreted as small (≥0.01), moderate (≥0.06), or large (≥0.14). Cohen’s d is reported for pairwise comparisons, interpreted as small (≥0.20), moderate (≥0.50), or large (≥0.80) [23].

Given the small sample size, a factorial multivariate analysis of variance (MANOVA; competition × time) was additionally applied to the grouped observations as an exploratory, hypothesis-generating step, providing a joint overview of the six variables prior to the variable-specific LMM and repeated-measures ANOVA analyses that constituted the primary inferential approach. Because MANOVA does not account for the repeated-measures structure of the design, its results are reported separately and interpreted descriptively only.

Because multivariate normality was not met, Pillai’s trace was used as the primary multivariate test statistic. Because this model does not account for the repeated-measures structure of the design, its results were interpreted solely as exploratory, and formal inference was based on LMMs and repeated-measures ANOVA. Type III factorial ANOVA served as the univariate follow-up to MANOVA.

To examine the effect of competition on each variable while taking advantage of the nested structure of the design, independent LMMs were fitted for each combination of dependent variable and cycle phase, with competition as a fixed effect and subject as a random intercept [variable ~ 1 + competition + (1|subject)], assuming a Gaussian distribution of residuals. Models were fitted by restricted maximum likelihood (REML) using the bobyqa optimizer, implemented via the GAMLj module (version 3.6.5) for jamovi (version 2.6; The jamovi project, Sydney, Australia), which calls the lmer function (lme4/lmerTest packages); confidence intervals for fixed-effect estimates were computed using the Wald method. For each model, residual diagnostics (residual-versus-predicted scatterplots and histograms of the subject-level random effects) were inspected to assess linearity, homoscedasticity, and the normality of the random effects. LMMs were chosen for their robustness when there are small samples, their independence from the sphericity assumption, and their ability to quantify stable interindividual variance. The intraclass correlation coefficient (ICC) was calculated as the proportion of total variance explained by the random subject effect based on the variance components of the fitted model. Degrees of freedom were estimated using the Satterthwaite approximation, and for each model, the F-statistic for the competition effect, the ICC, and the likelihood ratio test (LRT χ^2^) for the random component are reported, along with pairwise comparisons of estimated marginal means corrected using the Bonferroni method. Estimated marginal means are reported with 95% confidence intervals, and effect sizes with their corresponding 90% confidence intervals, throughout the Section 3. When the variance of the random intercept collapsed to zero (singular fit; ICC ≈ 0), the random component became uninterpretable, and the results for that variable–phase combination were derived solely from the corresponding repeated-measures ANOVA. Accordingly, LMMs were used as the primary models for between-competition comparisons within each phase, with competition as a fixed effect and subject as a random intercept. Repeated-measures ANOVA was used to assess temporal changes across the pre-competition, competition, and post-competition phases within each event.

The temporal dynamics within each competition were examined using repeated-measures ANOVA with phase (pre-competition, competition, and post-competition) as a within-subject factor.

Sphericity was not formally tested given the small sample size (*n* = 8), which limits the power of Mauchly’s test to detect departures from sphericity; the Greenhouse–Geisser correction was therefore applied systematically and conservatively to all repeated-measures ANOVAs with three levels, regardless of the outcome of any sphericity test, reporting the corresponding ε, the F statistic, η^2^p with its 90% CI, and pairwise comparisons with Bonferroni correction, accompanied by Cohen’s d for paired samples.

Intra-individual competitive performance across the three competitions was compared using the Friedman test, with post-hoc Durbin–Conover comparisons when appropriate. Kendall’s W was reported as an effect-size measure. Finally, exploratory associations between intra-individual competitive performance and the selected sleep variables were examined using Pearson’s r and Spearman’s ρ correlations computed on the pooled observations (*n* = 24; 8 swimmers × 3 competitions). As each swimmer contributed repeated observations across competitions, these bivariate correlations do not meet the independence assumption of standard correlation tests; they were therefore treated as purely descriptive and hypothesis-generating, without adjustment for multiple comparisons, and should be interpreted with caution.

Because each swimmer contributed SS values across all three competitions, associations among the pre-competition, competition, and post-competition SS were examined using repeated measures correlation (rmcorr) [24], which estimates the common within-subject association between two variables while appropriately accounting for the non-independence of observations from the same athlete. Given the limited statistical power associated with the small sample size, an exploratory individual-level outlier analysis was additionally conducted across all six variables and nine phase-by-competition combinations, using the standard 1.5×interquartile range (IQR) criterion. Claude (Claude Opus 5.5; Anthropic PBC, San Francisco, CA, USA) was used for assisting in the drafting and reporting of the statistical analysis section, specifically for the verification of calculated values, confidence intervals, and post hoc comparisons against the raw output from jamovi. The analyses were performed in Jamovi 2.7 [25]. Figures were generated in Python (version 3.13; Python Software Foundation, Wilmington, DE, USA).

## 3. Results

**Figure 1:**
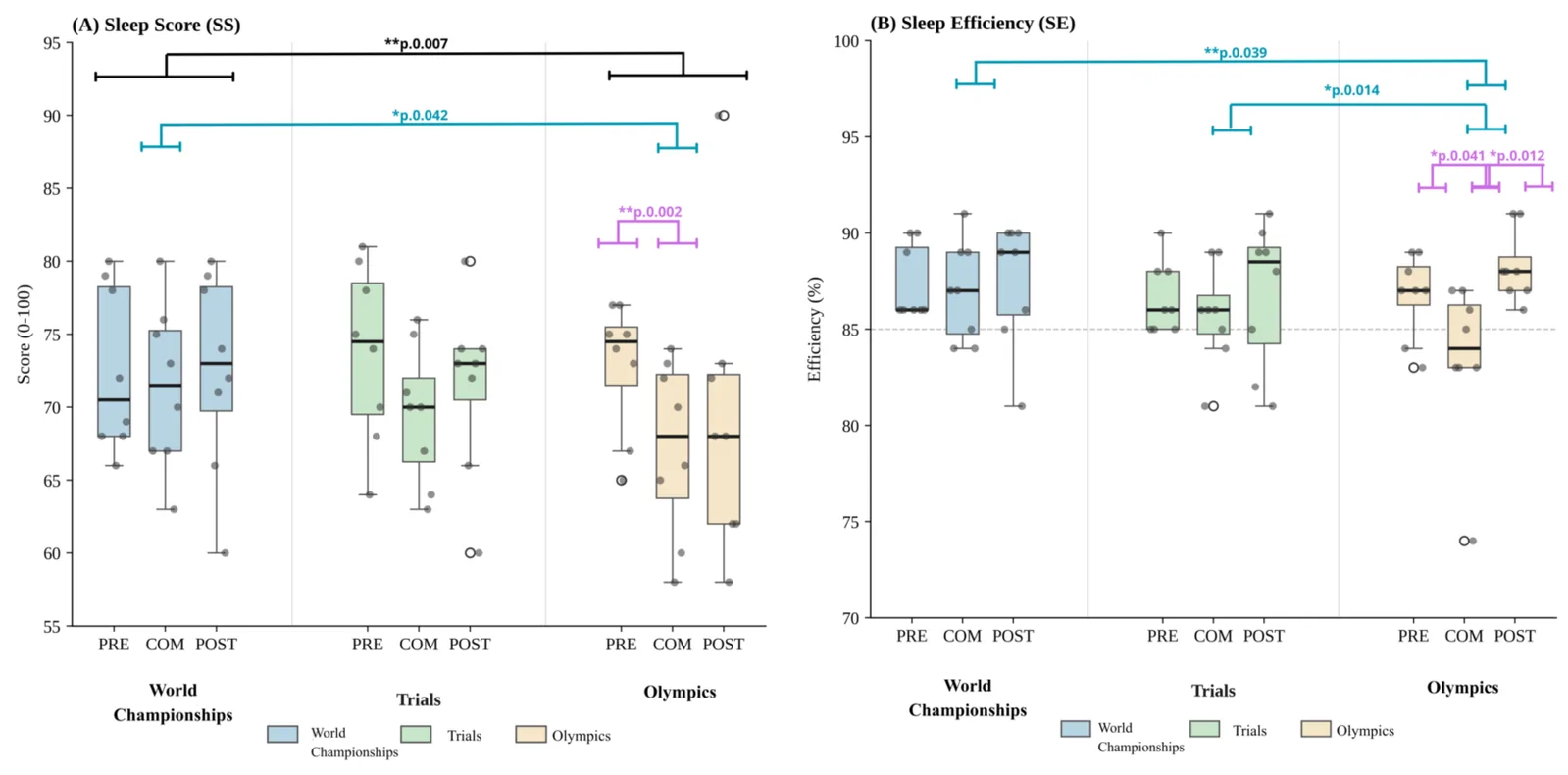
Sleep quality parameters by competition and stage of the competitive cycle: (**A**) the total sleep score (SS) and (**B**) sleep efficiency (SE). The boxes represent the median and interquartile range; the dots correspond to individual values (*n* = 8) and the hollow circles to outliers. The dashed line in panel (**B**) indicates the 85% clinical threshold. Black brackets indicate the overall effect of competition across events; blue brackets indicate pairwise between-competition comparisons within the same phase based on linear mixed models; and pink brackets indicate pairwise within-competition comparisons across the competitive phases based on repeated-measures analysis of variance. * *p* < 0.05; ** *p* < 0.01.

**Figure 2:**
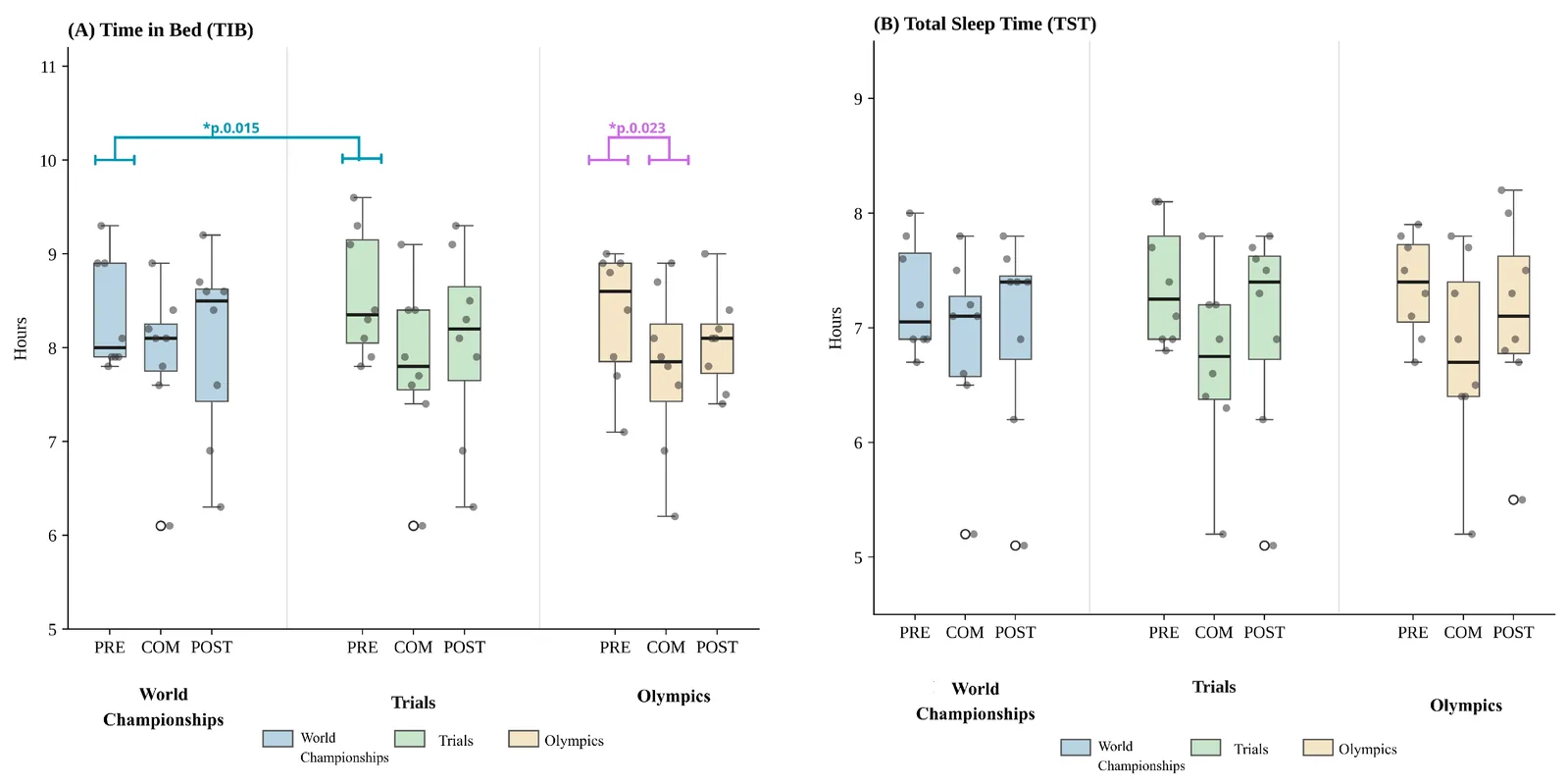
Sleep quantity parameters by competition and stage of the competitive cycle: (**A**) time in bed (TIB) and (**B**) total sleep time (TST). The boxes represent the median and interquartile range; the dots correspond to individual values (*n* = 8) and the hollow circles to outliers. Black brackets indicate the overall effect of competition across events; blue brackets indicate pairwise between-competition comparisons within the same phase based on linear mixed models; and pink brackets indicate pairwise within-competition comparisons across the competitive phases based on repeated-measures analysis of variance. * *p* < 0.05.

**Figure 3:**
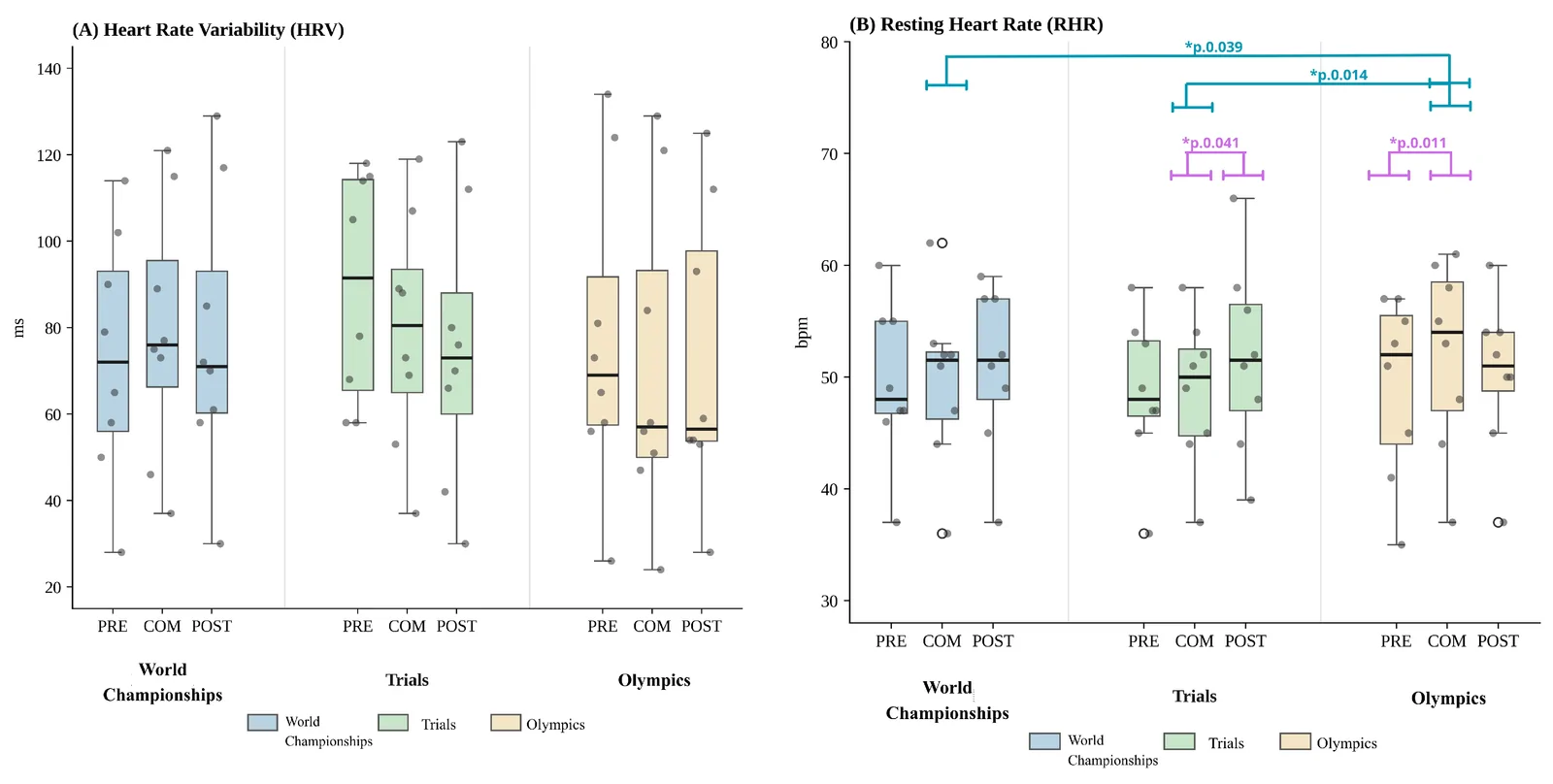
Autonomic physiological markers by competition and stage of the competitive cycle: (**A**) heart rate variability (HRV) and (**B**) resting heart rate (RHR). The boxes represent the median and interquartile range; the dots correspond to individual values (*n* = 8) and the empty circles to outliers. Black brackets indicate the overall effect of competition across events; blue brackets indicate pairwise between-competition comparisons within the same phase based on linear mixed models; and pink brackets indicate pairwise within-competition comparisons across the competitive phases based on repeated-measures analysis of variance.* *p* < 0.05.

Individual-level variability is represented in Figure 1, Figure 2 and Figure 3 through the overlaid raw data points (*n* = 8) within each boxplot. The exploratory outlier analysis identified sixteen outliers overall, half of which (8 of 16) were attributable to a single swimmer, who was flagged as an outlier in four of the six variables examined (SS, TIB, TST, and SE); this athlete consistently exhibited shorter and less efficient sleep than the remainder of the cohort across nearly all monitoring windows. Outlying values were therefore not evenly distributed across the cohort but concentrated predominantly in one individual.

### 3.1. Sleep Quality

Figure 1 presents the results of the parameters related to sleep quality.

For the SS, the inter-competition analysis revealed the main effect of competition was significant during the competition (F_(2,14)_ = 3.97; *p* = 0.043; η^2^p = 0.362; 90% CI 0.01–0.54; LRT χ^2^ = 16.19; *p* = 0.001; ICC = 0.732]. In the LMM, swimmers recorded lower scores at the Olympic Games than at the World Championships (t_(14)_ = 2.81, *p* = 0.042; d = 1.69; 95% CI 0.56–2.78), with an estimated mean of 67.2 (95% CI 63.1–71.4; median = 68.0, IQR = 63.75–72.25) and 71.1 (95% CI 66.9–75.3; median = 71.5, IQR = 67.0–75.25) points, respectively. In the repeated-measures ANOVA, there was a significant difference between the Olympics Games and the World Championships during the competition phase (Bonferroni-adjusted *p* = 0.007). There were no significant effects in the pre-competition phase (F_(2,14)_ = 0.606; *p* = 0.559; η^2^p = 0.080; 90% CI 0.00–0.26; ICC = 0.815) or in the post-competition phase (F_(2,14)_ = 2.55; *p* = 0.114; η^2^p = 0.267; 90% CI 0.00–0.47; ICC = 0.576). At the intra-competition level, the SS decreased significantly between the pre-competition and competition phases at the Olympic Games (*p* = 0.002; d = 2.03; 95% CI 0.76–3.26), with no significant changes observed at the World Championships (ε= 0.58; F_(1.16,8.12)_ = 0.221; *p* = 0.805; η^2^p = 0.031; 90% CI 0.000–0.155) or at the Trials (ε= 0.90; F_(1.80,12.60)_ = 2.14; *p* = 0.154; η^2^p = 0.234; 90% CI 0.000–0.435).

In the LMM, the main effect of competition was significant for SE during the competition phase (F_(2,14)_ = 8.58; *p* = 0.004; η^2^p = 0.551; 90% CI 0.16–0.69; LRT χ^2^ = 15.05; ICC = 0.595), with significantly lower values at the Olympic Games compared with the World Championships (*p* = 0.039; d = 1.30; 95% CI 0.31–2.23) and the Trials (*p* = 0.014; d = 0.85; 95% CI 0.01–1.64), with an estimated mean of 82.9% (95% CI 80.4–85.3; median = 84.0%, IQR = 83.0–86.25%), 87.0% (95% CI 84.5–89.5; median = 87.0%, IQR = 84.75–89.0%), and 85.8% (95% CI 83.3–88.2; median = 86.0%, IQR = 84.75–86.75%), respectively. There were no differences for the pre-competition (F_(2,14)_ = 1.35; *p* = 0.292; η^2^p = 0.162; 90% CI 0.00–0.36) and post-competition (F_(2,14)_ = 1.20; *p* = 0.332; η^2^p = 0.146; 90% CI 0.00–0.35) phases. At the intra-competition level, the time mean effect was significant at the Olympic Games (ε = 0.67; F(1.35,9.44) = 20.72, *p* < 0.001; η^2^p = 0.747; 90% CI 0.36–0.84), with a significant decrease between the pre-competition and competition phases (*p* = 0.041; d = 1.64; 95% CI 0.53–2.71) and subsequent recovery in the post-competition phase (*p* = 0.012; d = 1.73; 95% CI 0.58–2.83). In contrast, there were no significant changes at the Trials (ε= 0.74; F_(1.48,10.36)_ = 0.921; *p* = 0.399; η^2^p = 0.116; 90% CI 0.00–0.36) and at the World Championships (ε= 0.99; F_(1.98,13.86)_ = 0.185; *p* = 0.83; η^2^p = 0.026; 90% CI 0.00–0.14).

### 3.2. Sleep Quantity

Figure 2 presents the results of the parameters related to sleep duration.

In the LMM, TIB did not differ significantly across competitions during the competition phase (F_(2,14)_ = 0.912; *p* = 0.424; η^2^p = 0.115; 90% CI 0.00–0.31; LRT χ^2^ = 5.198; *p* = 0.158; ICC = 0.430), and no post hoc comparison reached significance after Bonferroni correction. In addition, TIB did not differ across the competitions for the post-competition phase (F_(2,14)_ = 0.0161; *p* = 0.984; η^2^p = 0.002; 90% CI 0.00–0.00; ICC = 0.583) and the pre-competition phase (F_(2,14)_ = 2.46; *p* = 0.121; η^2^p = 0.260; 90% CI 0.00–0.46; LRT χ^2^ = 4.867; *p* = 0.088; ICC = 0.848). Moreover, none of the LMM-based pairwise comparisons reached significance after Bonferroni correction for either phase. However, a between-competition comparison of the pre-competition phase using repeated-measures ANOVA indicated that TIB was significantly higher at the Trials (median = 501 min, IQR = 483–549 min) than at the World Championships (median = 480 min, IQR = 474–534 min) (*p* = 0.015; d = 1.98; 95% CI 0.73–3.19). At the intra-competition level, TIB varied significantly across phases at the Trials (ε = 0.97; F_(1.93,13.51)_ = 4.67, *p* = 0.030; η^2^p = 0.400; 90% CI 0.03–0.58), although no individual pairwise comparison reached significance after Bonferroni correction. No significant intra-event changes were observed at the World Championships (ε = 0.70; F_(1.40,9.81)_ = 0.70, *p* = 0.471; η^2^p = 0.091; 90% CI 0.00–0.34) or the Olympic Games (ε = 0.61; F_(1.22,8.55)_ = 2.22, *p* = 0.172; η^2^p = 0.241; 90% CI 0.00–0.51), despite the significant pre-to-competition reduction reported below. At the Olympic Games, there was a significant reduction in TIB between the pre-competition (median = 516 min, IQR = 471–534 min) and competition (median = 471 min, IQR = 445–495 min) phases (*p* = 0.023; d = 1.31; 95% CI 0.32–2.25).

In the LMM, TST did not differ across the competitions for any phase (pre-competition: F_(2,14)_ = 0.726; *p* = 0.501; η^2^p = 0.094; 90% CI 0.00–0.28; ICC = 0.756; competition: F_(2,14)_ = 0.985; *p* = 0.398; η^2^p = 0.123; 90% CI 0.00–0.32; ICC = 0.772; post-competition: F_(2,14)_ = 0.558; *p* = 0.584; η^2^p = 0.074; 90% CI 0.00–0.25; ICC = 0.740), with stable interindividual variance confirmed by the LRT for the random component (competition: LRT χ^2^ = 16.69; *p* < 0.001; post-competition: LRT χ^2^ = 14.61; *p* = 0.002). The mean TST during the competition phase was 402 min (median = 405 min, IQR = 383–432 min) for the Trials, 413 min (median = 426 min, IQR = 395–437 min) for the World Championships, and 407 min (median = 402 min, IQR = 384–444 min) for the Olympic Games. There were no significant differences in TST across the phases within any of the three competitions (World Championships: F_(2,14)_ = 1.50; *p* = 0.257; η^2^p = 0.176; 90% CI 0.000–0.376; Trials: ε = 0.87; F_(1.73,12.11)_ = 3.66, *p* = 0.052; η^2^p = 0.344; 90% CI 0.000–0.529; Olympic Games: ε = 0.89; F_(1.77,12.39)_ = 3.01; *p* = 0.081; η^2^p = 0.301; 90% CI 0.000–0.494).

### 3.3. Autonomic Physiological Markers

Figure 3 presents the results of the parameters related to autonomic physiological markers.

In the LMMs, HRV did not differ significantly across the competitions for any phase (pre-competition: F_(2,14)_ = 0.629; *p* = 0.548; η^2^p = 0.082; 90% CI 0.00–0.26; competition: F_(2,14)_ = 2.11; *p* = 0.158; η^2^p = 0.232; 90% CI 0.00–0.43; post-competition: F_(2,14)_ = 1.26; *p* = 0.315; η^2^p = 0.153; 90% CI 0.00–0.35], with high ICC values for the pre-competition (LRT χ^2^ = 32.46; *p* < 0.001; ICC = 0.921), competition (LRT χ^2^ = 32.13; *p* < 0.001; ICC = 0.918), and post-competition (LRT χ^2^ = 40.96; *p* < 0.001; ICC = 0.954] phases. The estimated mean during the competition phase was 79.4 ms (95% CI 53.6–105.2; median = 80.5 ms, IQR = 65.0–93.5 ms) for the Trials, 79.1 ms (95% CI 53.3–104.9; median = 76.0 ms, IQR = 66.25–95.5 ms) for the World Championships, and 71.2 ms (95% CI 45.5–97.0; median = 57.0 ms, IQR = 50.0–93.25 ms) for the Olympic Games. At the intra-event level, repeated-measures ANOVA indicated no significant changes across phases at the Trials (F_(2,14)_ = 1.20; *p* = 0.329; η^2^p = 0.147; 90% CI 0.000–0.347), the World Championships (F_(2,14)_ = 0.27; *p* = 0.770; η^2^p = 0.037; 90% CI 0.000–0.174), or the Olympic Games (F_(2,14)_ = 2.64; *p* = 0.107; η^2^p = 0.274; 90% CI 0.000–0.471), although the pre-competition-to-competition comparison at the Olympic Games approached statistical significance (t_(7)_ = 2.85; *p* = 0.075). At the inter-competition level, there were no differences for any event.

RHR differed significantly across the competitions during the competition phase (F_(2,14)_ = 6.55; *p* = 0.010; η^2^p = 0.483; 90% CI 0.09–0.64; LRT χ^2^ = 35.34; *p* < 0.001; ICC = 0.931), with an estimated mean of 48.8 bpm (95% CI 42.7–54.8; median = 50.0 bpm, IQR = 44.75–52.5 bpm) for the Trials, 49.3 bpm (95% CI 43.2–55.3; median = 51.5 bpm, IQR = 46.25–52.25 bpm) for the World Championships, and 52.0 bpm (95% CI 46.0–58.0; median = 54.0 bpm, IQR = 47.0–58.5 bpm) for the Olympic Games. Pairwise comparisons revealed higher values at the Olympic Games compared with the Trials (t_(14)_ = 3.36; difference = 3.25 bpm; *p* = 0.014; d = 1.06; 95% CI 0.16–1.92) and the World Championships (t_(14)_ = 2.85; difference = 2.75 bpm; *p* = 0.039; d = 0.89; 95% CI 0.04–1.69). There were no significant differences for the pre-competition phase (F_(2,14)_ = 0.253; *p* = 0.780; η^2^p = 0.035; 90% CI 0.00–0.17; ICC = 0.880) or the post-competition phase (F_(2,14)_ = 0.779; *p* = 0.478; η^2^p = 0.100; 90% CI 0.00–0.29; ICC = 0.89). At the intra-competition level, RHR varied significantly across phases at the Trials (ε = 0.89; F_(1.78,12.48)_ = 5.21; *p* = 0.025; η^2^p = 0.427; 90% CI 0.04–0.60), with higher values during the post-competition phase (median = 51.5 bpm, IQR = 47.0–56.5 bpm) than during the pre-competition phase (median = 48.0 bpm, IQR = 46.5–53.25 bpm) (t_(7)_ = –3.28; difference = 3.125 bpm; *p* = 0.041; d = 1.16; 95% CI 0.22–2.05). No significant intra-event changes were observed at the World Championships (ε = 0.86; F_(1.71,12.00)_ = 2.54; *p* = 0.125; η^2^p = 0.266; 90% CI 0.00–0.48). At the Olympic Games, the omnibus effect across phases did not reach significance (ε = 0.60; F_(1.21,8.44)_ = 1.78; *p* = 0.221; η^2^p = 0.203; 90% CI 0.00–0.48), although RHR was higher during the competition phase (median = 54.0 bpm, IQR = 47.0–58.5 bpm) than during the pre-competition phase (median = 52.0 bpm, IQR = 44.0–55.5 bpm) (t_(7)_ = –4.25; difference = 2.75 bpm; *p* = 0.011; d = 1.50; 95% CI 0.44–2.51).

### 3.4. Multivariate and Exploratory Analysis

Table 1 presents the results of the multivariate and exploratory analysis.

The exploratory factorial MANOVA using Pillai’s trace did not reveal a significant competition effect (V = 0.254; F_(16,114)_ = 1.035; *p* = 0.426), time effect (V = 0.387; F_(16,114)_ = 1.709; *p* = 0.054), or competition × time interaction (V = 0.417; F_(32,236)_ = 0.858; *p* = 0.690). Given the violation of multivariate normality and the fact that the exploratory MANOVA did not account for the repeated-measures structure of the study, these findings were interpreted descriptively. Exploratory follow-up univariate analyses (Table 1) showed that the effect of time was significant only for SE (F_(2,63)_ = 4.512; *p* = 0.015; η^2^p = 0.125); the remaining variables did not reach statistical significance (SS: F_(2,63)_ = 2.504; *p* = 0.090; TIB: F_(2,63)_ = 2.511; *p* = 0.089; TST: F_(2,63)_ = 2.444; *p* = 0.095; HRV: F_(2,63)_ = 0.522; *p* = 0.596; RHR: F_(2,63)_ = 0.352; *p* = 0.705).

### 3.5. Competitive Performance

Table 2 presents the results of competitive performance by competition.

At the World Championships, 5 of the 8 athletes (62.5%) recorded their worst relative performance (Level 3). At the Trials, 5 of the 8 athletes (62.5%) achieved their best performance (Level 1). At the Olympic Games, 4 of the 8 athletes (50%) achieved an intermediate performance (Level 2). Because Fisher’s exact test and the chi-square test treat the 24 observations (8 swimmers × 3 competitions) as independent, whereas each swimmer contributed data to all three competitions, a Friedman test was used instead, ranking performance within each swimmer to properly account for the repeated-measures structure. The Friedman test did not indicate a significant association between competition and intra-individual performance (χ^2^(2) = 2.40; *p* = 0.301; Kendall’s W = 0.150), and Durbin–Conover pairwise comparisons showed no significant differences between any pair of competitions (Mundial vs. Trials: *p* = 0.138; Mundial vs. Olympic Games: *p* = 0.445; Trials vs. Olympic Games: *p* = 0.445).

Exploratory bivariate correlation analyses did not detect a significant association between intra-individual qualitative performance and the pre-competition SS (Pearson’s r = −0.120, 95% CI: −0.50 to 0.30, *p* = 0.576; Spearman’s ρ = −0.140, *p* = 0.513), the competition SS (Pearson’s r = 0.049, 95% CI: −0.36 to 0.44, *p* = 0.821; Spearman’s ρ = 0.044, *p* = 0.837), or the post-competition SS (Pearson’s r = 0.316, 95% CI: −0.10 to 0.63, *p* = 0.133; Spearman’s ρ = 0.329, *p* = 0.116). Because each swimmer contributed SS values across all three competitions, associations among the pre-competition, competition, and post-competition SS were instead examined using repeated measures correlation, which appropriately accounts for the non-independence of observations within athletes. No significant repeated measures correlation was detected between the pre-competition and competition SS (r_rm = 0.048, 95% CI: −0.44 to 0.52, *p* = 0.854), between the pre-competition and post-competition SS (r_rm = −0.376, 95% CI: −0.73 to 0.13, *p* = 0.137), or between the post-competition and competition SS (r_rm = 0.138, 95% CI: −0.37 to 0.58, *p* = 0.597). Given the small sample size and the correspondingly wide confidence intervals, non-significant correlations should not be interpreted as evidence of the absence of an association.

Expressed continuously, the percentage change in official time was, on average, −0.96% ± 1.41% between the World Championships and the Trials (range: −2.87% to +1.29%), indicating a modest overall improvement consistent with the high proportion of athletes reaching Level 1 at the Trials. Between the Trials and the Olympic Games, the mean change was +2.04% ± 3.58% (range: −0.52% to +8.16%), reflecting an overall slowdown, most pronounced in the two open-water swimmers (+7.35% and +8.16%), consistent with the higher proportion of athletes recording an intermediate or worst relative performance at the Olympic Games.

## 4. Discussion

This study conducted a longitudinal follow-up of sleep and autonomic activity in eight Olympic swimmers across three international competitions within the same Olympic cycle, under real-world conditions. The main finding is that the sporting context had a variable influence on rest and autonomic regulation, and that this effect cannot be explained solely by the physical demands of the event.

The SS declined at the start of competition and partially recovered afterward, suggesting an acute, situation-dependent response rather than chronic, cumulative fatigue. Reviews of sleep interventions in elite sports have identified sleep hygiene as the most effective measure, ahead of supplementation or light therapy [26]. In the present study, average SE during the competition phase of the Olympic Games dropped to 82.9%, below the commonly used reference threshold of 85% adopted in wearable-based sleep research [10]; this value functions as a general benchmark rather than a clinical diagnostic criterion, and should be interpreted accordingly given that measurements were obtained using a consumer wearable device in elite athletes rather than under clinical polysomnographic conditions. This decline did not occur at the World Championships (87.0%), despite its international status, nor at the Trials (85.8%), despite their strict qualification standards for the Olympic Games. Thus, the Olympic Games were the only competition in this sample in which SE fell below the 85% threshold; even at this lowest point, average sleep efficiency remained close to recommended levels, and across the remainder of the monitoring period sleep duration and quality were consistently at or above recommended thresholds.

High psychological stress in Olympic athletes has been previously associated with poorer sleep in other cohorts [27]. The fact that the World Championships in Doha did not produce the same effect is consistent with the hypothesis that competitive hierarchy alone does not explain the decline, and that the Olympic setting specifically may introduce a distinct allostatic load [28]; this interpretation, however, rests on external evidence rather than on direct assessment of psychological stress within the present sample.

This psychosocial component was compounded by the environmental conditions of Paris 2024. Accommodation conditions differed by competition: athletes stayed in hotels during the World Championships and the Trials, and in the Olympic Village during the Olympic Games. No illness was reported among participants before, during, or after any of the three events. The absence of air conditioning in the Olympic Village, during a summer with high temperatures exceeding 35 °C, may have contributed to an unfavorable nighttime environment; heat exposure is associated with increased wakefulness and reduced slow-wave and REM sleep [29]. It had already been predicted that high temperatures during the Paris Games would hinder the drop in core body temperature necessary to initiate and consolidate deep sleep [30], a mechanism whose thermal dependence has been well established [31]. Such sleep disruption may compromise the consolidation of motor memory and technical skills [2], which are critical in swimming. The combination of morning and evening sessions across the competition has similarly been shown to fragment the sleep–wake cycle in comparable elite swimming cohorts [32].

TIB provides additional insight into how swimmers coped with increasing demands. During the pre-competition phase, TIB was higher at the Trials than at the World Championships (difference ≈ 13.5 min), consistent with previous findings of increased TIB in the days leading up to competition [33]. The most significant change was observed at the Olympics Games: TIB decreased between the pre-competition and competition phases (difference ≈ 34.5 min), reversing the protective pattern observed at the Trials. The Olympic environment was accompanied by poorer sleep quality and reduced time available for sleep, potentially reflecting the confluence of logistical obligations [34], afternoon or evening competition schedules [35], and pre-competition arousal, with comparable sleep changes reported around race sessions in elite swimming cohorts [32].

TST during the pre-competition phase was slightly lower at the World Championships than at the Trials (difference ≈ 7.5 min). This pattern—a higher TIB without a proportional increase in sleep obtained—has also been described in other elite swimming cohorts across different training and competitive phases [36]. In Paris, higher nighttime temperatures may have exacerbated this gap by hindering the thermoregulation necessary for slow-wave sleep [31]; as ambient temperature was not directly recorded in the present study, this interpretation is inferred from the known environmental conditions of the 2024 Games rather than from measured data. Both station-based observations and climatological records for the corresponding periods indicate that the World Championships (Doha, February 2024; typical daily highs of approximately 23–24 °C) and the Trials (Palma de Mallorca, 18–22 June 2024; official meteorological records, AEMET, report a monthly maximum of 35.2 °C recorded later in the month, with conditions during the competition window remaining below 30 °C) took place under comparatively mild thermal conditions, whereas the Paris 2024 Olympic Games coincided with a documented summer heatwave exceeding 35 °C. Sleep hygiene has been proposed as a countermeasure in these environments [37].

RHR rose significantly during the competition phase of the Olympic Games, and this increase was circumstantial: it did not differ between competitions either before or after the event. This temporal specificity is informative because it rules out a baseline alteration in autonomic status and positions the response as an acute reaction to the period of maximum exertion. RHR is a recognised indicator of accumulated load and recovery status [11]. HRV, on the other hand, did not show statistically significant differences across the competitive phases; in the absence of statistical significance and given the limited power associated with the sample size, this pattern remains inconclusive. Caution is warranted when considering this variable: its high intra-individual stability, reflected in the high ICCs, implies that each athlete has a unique autonomic signature and that changes can only be interpreted relative to the individual baseline, as noted in the framework for longitudinal monitoring of HRV in elite athletes [11]. This characteristic, combined with the small sample size, limits the ability to detect group differences.

The divergence in the intra-competition kinetics of RHR between the Olympic Games and the Trials suggests different underlying mechanisms. At the Olympic Games, the increase occurred during the competition itself, a timing consistent with elevated psychological stress previously documented in Olympic athletes [27]. At the Trials, by contrast, the increase occurred later, a pattern more consistent with the classic kinetics of physiological HRV accumulated following competitive exertion [38]. Taken together, we interpret the elevation in RHR at the Olympic Games as a pattern consistent with increased autonomic strain, given that insufficient sleep is in itself an autonomic stressor [39]. This convergence of markers reinforces the importance of not interpreting any of them in isolation, but rather integrating them with subjective and external load data for a reliable assessment of readiness [40].

When accounting for the non-independence of repeated observations within swimmers, no statistically significant associations were found among sleep scores across the different competitive phases or between sleep metrics and intra-individual competitive performance, consistent with previous research in swimmers reporting no significant correlation between subjective sleep quality and swim time [41]. Overall, swimmers maintained generally adequate sleep duration and quality across the study period, with only modest changes observed during competition. Performance in elite swimming is multifactorial [42] and may be influenced by technique, race tactics [43], physical condition, and competitive activation [32]. Added to this is a statistical consideration: a sample of eight athletes provides limited statistical power to detect small correlations, so the absence of statistical significance does not equate to the absence of a relationship. The existing literature supports this complexity: nocturnal HRV is used as a tool for monitoring recovery status rather than as a predictor of performance [11], and accumulated fatigue prior to tapering—associated with poorer sleep—has been linked to lower performance gains following a reduction in training load among elite swimmers [44]. Even the subjective perception of inadequate rest is associated with poorer mood during competitive periods [45].

## 5. Strengths and Limitations

The main strength of this study is the longitudinal, intra-individual monitoring of eight Olympic-level swimmers across three competitions of varying importance within the same Olympic cycle. As noted in Section 2.1, this cohort represents a substantial proportion of the national Olympic swimming population, an elite and inherently difficult-to-access group that is rarely studied under real-world, ecologically valid conditions across an entire Olympic year. However, several limitations must be acknowledged. First, sleep and autonomic markers were assessed exclusively through a consumer wearable device, without subjective questionnaires, baseline (off-season) measurements, or control for potentially relevant confounders such as circadian disruption from travel, coaching or family support, or personal circumstances. The explanations proposed in the Discussion regarding psychosocial stress and environmental conditions are supported by prior evidence in other Olympic cohorts but remain unconfirmed as direct mechanisms within the present sample, as psychological stress was not directly measured in this study. The role of ambient temperature is supported by external literature specific to the Paris 2024 context [30], although within-study comparative temperature data across the three events, and athletes’ heat acclimatization status, were not collected. Second, the small sample size substantially limits statistical power, particularly for the exploratory correlational and categorical analyses; non-significant findings should not be interpreted as evidence of the absence of an effect, and this constraint reflects the scarcity of the population under study rather than a methodological shortcoming. Third, the six-month interval between the World Championships and the Trials/Olympic Games allowed for individual variation in training load and tapering strategies that was not controlled for. Future studies would benefit from incorporating subjective wellbeing and sleep-quality questionnaires alongside objective wearable data to provide a more complete assessment of an athlete’s readiness.

## 6. Conclusions

The results present a combined phenomenon rather than the sum of isolated effects. SE was found to be sensitive to the timing within the competitive cycle, while RHR responded to the type of competition, and both deteriorated simultaneously and selectively in the Olympic setting. These findings suggest that an athlete’s condition during high-level competition cannot be assessed based on a single indicator, and that the concurrent changes observed in sleep and autonomic regulation during the Olympic Games warrant further investigation with direct measures of the underlying contextual factors. The integration of objective sleep markers and nocturnal autonomic recordings provides a more complete picture of the physiological toll imposed by an event of the magnitude of the Olympic Games than either measure alone.

## Figures and Tables

**Table 1:** Univariate analysis examining the effect of time on sleep variables and autonomic markers.

Variable	SC	df	MS	F	*p*
SS (0–100)	172.75	2	86.38	2.504	0.090
SE (%)	70.778	2	35.38	4.512	0.015 *
TIB (h)	3.098	2	1.544	2.511	0.089
TST (h)	2.662	2	1.331	2.444	0.095
HRV (ms)	1018.69	2	509.35	0.522	0.596
RHR (bpm)	38.58	2	19.29	0.352	0.705

Note. df, degrees of freedom; HRV, heart rate variability; MS, mean square; RHR, resting heart rate; SC, sum of squares; SE, sleep efficiency; SS, sleep score; TIB, time in bed; TST, total sleep. * *p* < 0.05.

**Table 2:** Mean ranks of intra-individual competitive performance by competition (Friedman test).

Competition	Mean Rank
World Championships	1.62
Trials	2.00
Olympic Games	2.38

Note. Ranks (1 = fastest; 3 = slowest) were computed within each swimmer across their three competitions and averaged across swimmers (*n* = 8).

## Data Availability

The data presented in this study are available from the corresponding author upon reasonable request, subject to institutional and privacy restrictions.

## Abbreviations

The following abbreviations are used in this manuscript:

ANOVA	Analysis of variance
bpm	Beats per minute
CI	Confidence interval
ICC	Intraclass correlation coefficient
LMM	Linear mixed model
LRT	Likelihood ratio test
MANOVA	Multivariate analysis of variance
SE	Sleep efficiency
SS	Total sleep score
HRV	Heart rate variability
PSG	Polysomnography
RHR	Resting heart rate
TIB	Time in bed
TST	Total sleep time
